# Supplementary figures and images for: HPV upregulates MARCHF8 ubiquitin ligase and inhibits apoptosis by degrading the death receptors in head and neck cancer
Source: PLoS Pathog. 2023 Mar 3;19(3):e1011171. doi: 10.1371/journal.ppat.1011171 (PMC10016708; doi:10.1371/journal.ppat.1011171)

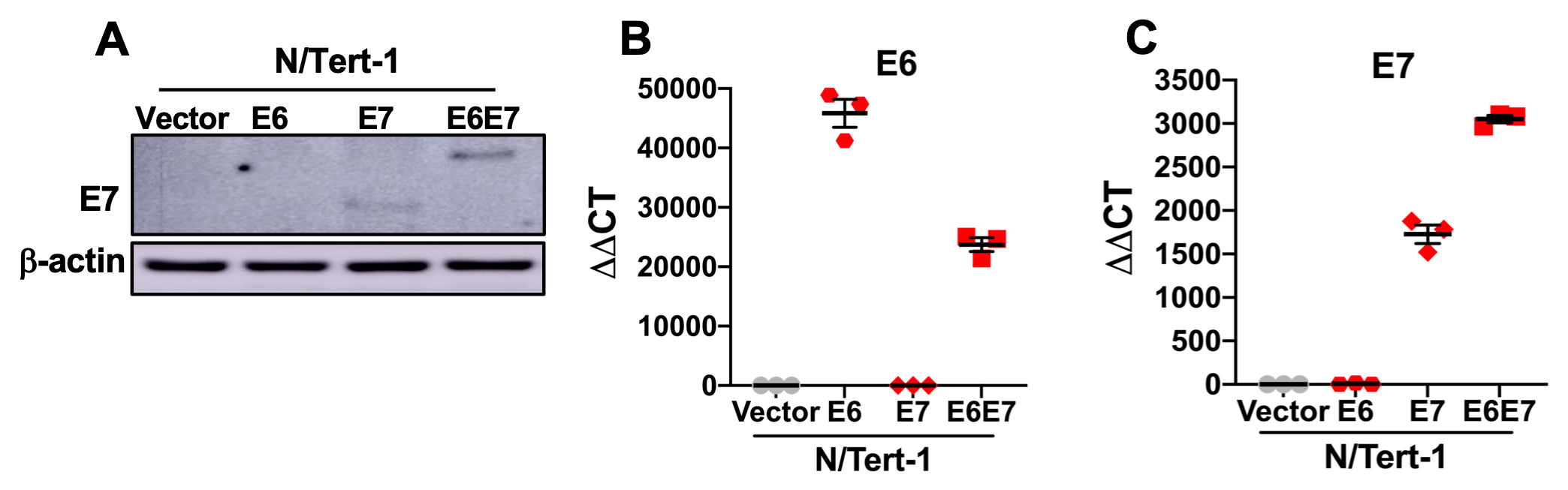

Supplement: S1 Fig — The HPV16 E7 protein levels were determined in N/Tert-1 cells and N/Tert-1 cells expressing HPV16 E6, E7, or E6 and E7 (E6E7) using western blotting (A). β-actin was used as a loading control. The size of HPV16 E7 in N/Tert-1 E7 cells is about 22 kDa because the protein is fused to the HA tag, while the size of HPV16 E7 in N/Tert-1 E6E7 cells is about 17 kDa because the protein is untagged. Total RNA was extracted from N/Tert-1 containing an empty vector and N/Tert-1 cells expressing HPV16 E6, E7, or E6 and E7 (E6E7). The HPV16 E6 (B) and E7 (C) mRNA expression levels were quantified by RT-qPCR. The data shown are normalized by the GAPDH mRNA level as an internal control. All experiments were repeated at least three times, and the data shown are means ± SD. (TIFF) [file ppat.1011171.s001.tiff]

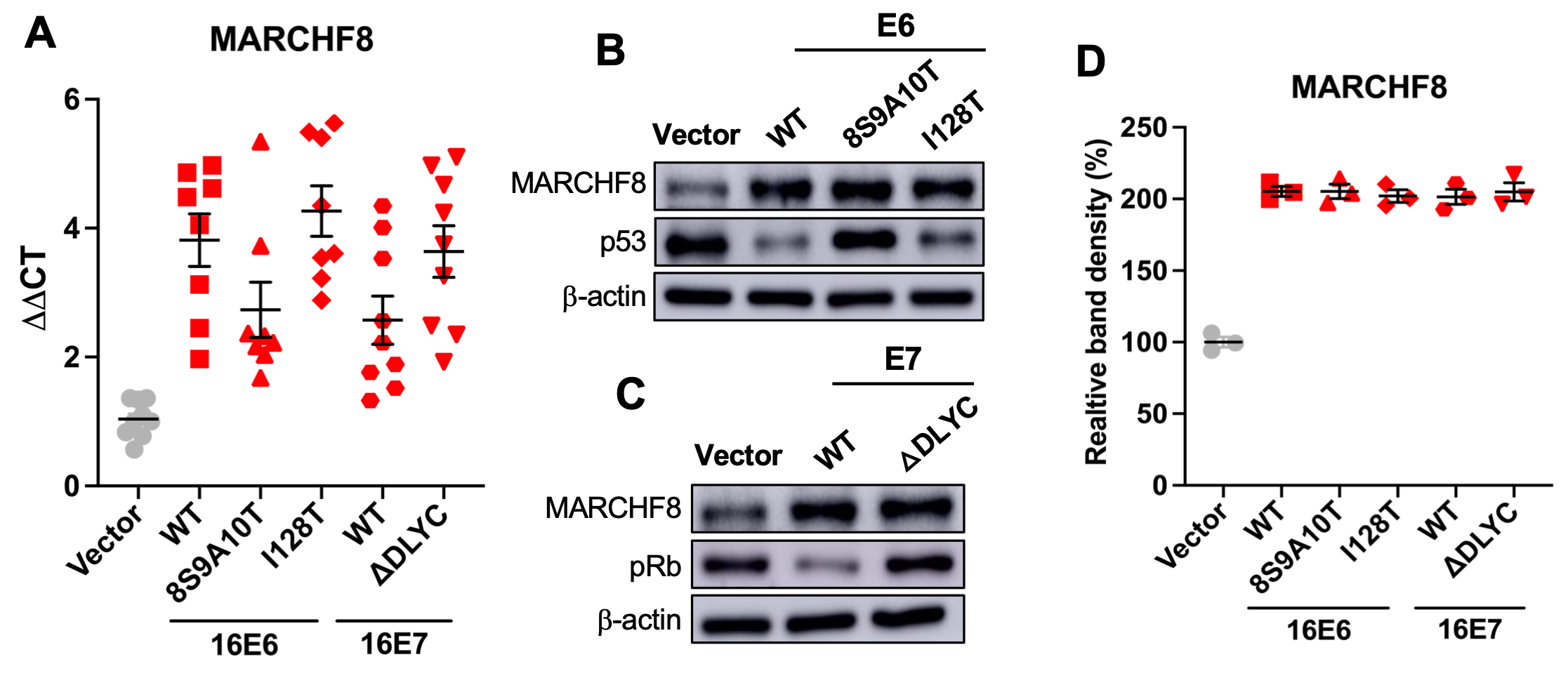

Supplement: S2 Fig — MARCHF8 mRNA (A) and protein (B—D) were determined in N/Tert-1 cells and N/Tert-1 cells expressing HPV16 E6, E6 8S9A10T, and E6 I128T (A and B) or E7 and E7 ΔDLYC (A and C) using RT-qPCR and western blotting, respectively. RT-qPCR was performed using total RNA extracted from N/Tert-1 cells, and the data shown are normalized by the GAPDH mRNA level as an internal control (A). Western blotting of E6 and p53 (B) or E7 and pRb (C) was performed using N/Tert-1 cell lysates with β-actin as a loading control. The relative band density was quantified using Image (D). All experiments were repeated at least three times, and the data is shown as mean ± SD. (TIFF) [file ppat.1011171.s002.tiff]

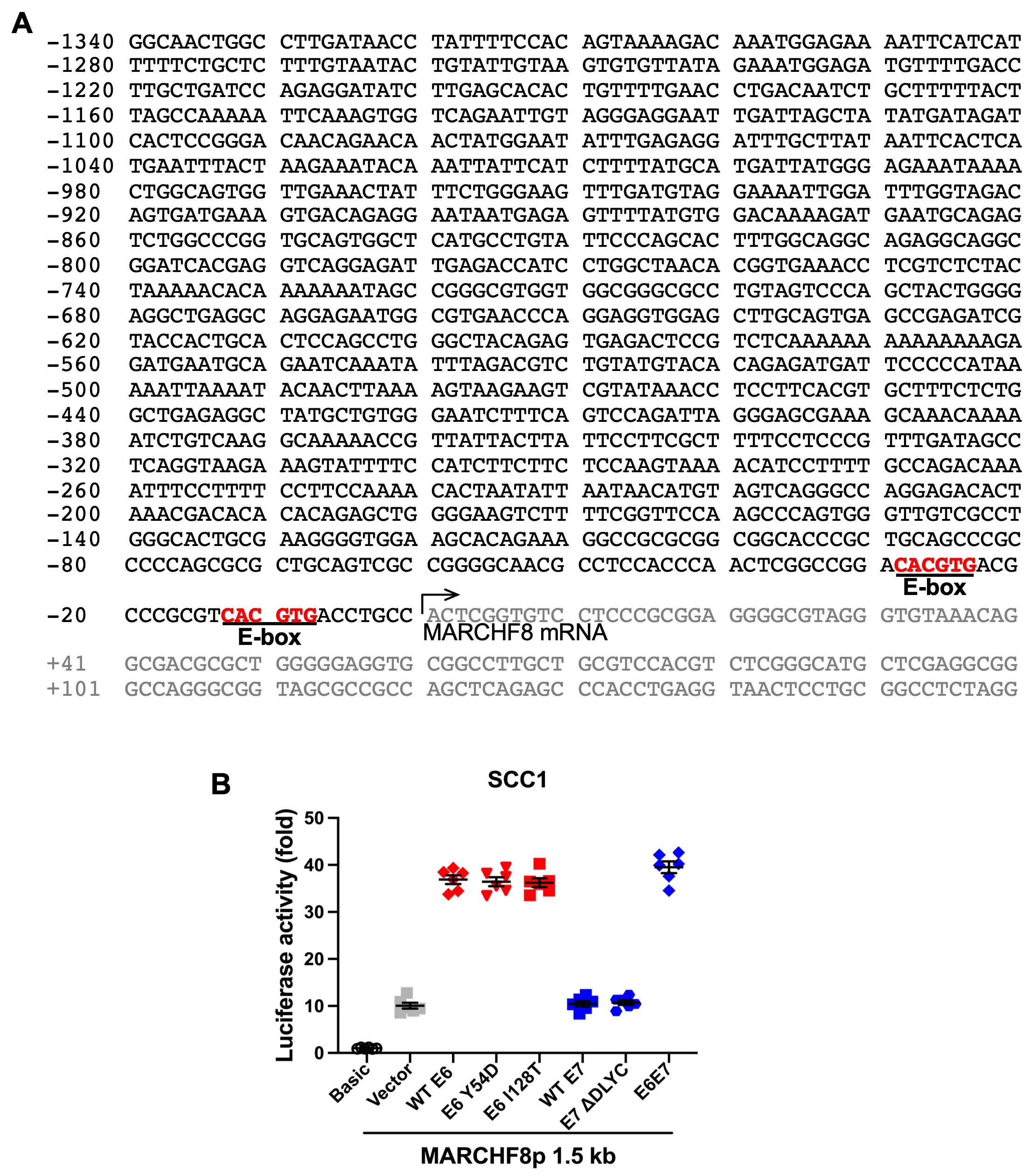

Supplement: S3 Fig — The map of the extended MARCHF8 promoter region (-1340 to +160) is shown with the positions of two E-boxes (red) and transcription start sequence (TSS, gray) (A). The promoter-reporter construct (-1340 to +160) was transfected into HPV- (SCC1) cells and cotransfected with plasmids expressing wildtype E6, E6 Y54D, E6 I128T deficient in E6AP binding, wildtype E7, or E7 ΔDLYC deficient in pRb binding (B). Luciferase activity was measured 48 h post transfection. Representative data from three independent experiments are shown as a fold change relative to the empty pGL4.2 vector (Basic). P values were determined by Student’s t-test. *p < 0.05, **p < 0.01, ***p < 0.001. (TIFF) [file ppat.1011171.s003.tiff]

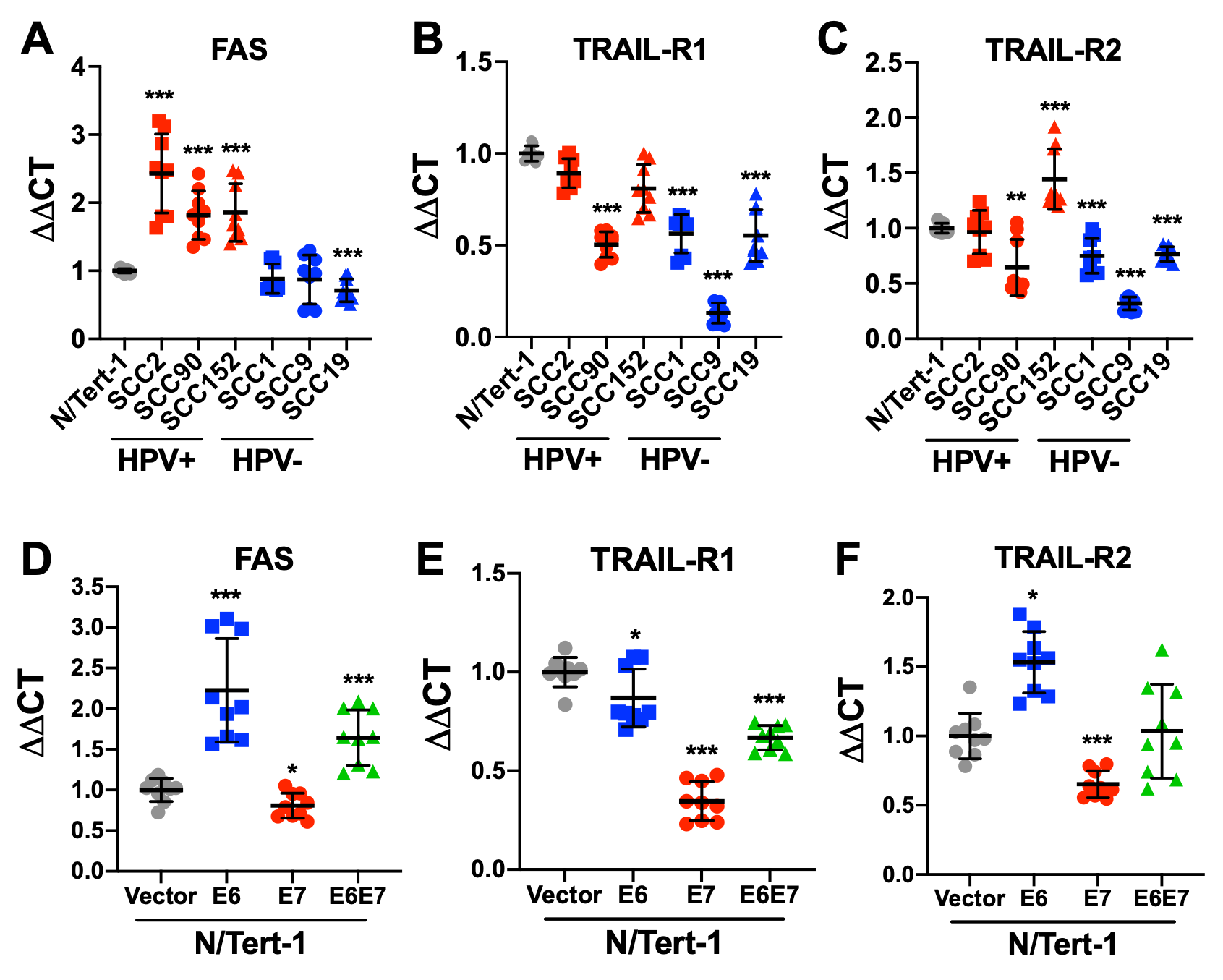

Supplement: S4 Fig — The FAS, TRAIL-R1, and TRAIL-R2 mRNA expression levels in normal (N/Tert-1), HPV+ HNC (SCC2, SCC90, and SCC152), and HPV- HNC (SCC1, SCC9, and SCC19) cells (A-C) and N/Tert-1 cells expressing HPV16 E6, E7, or E6 and E7 (D-F) were quantified by RT-qPCR. The data shown are normalized by the GAPDH mRNA level as an internal control. All experiments were repeated at least three times, and the data shown are means ± SD. P values were determined by Student’s t-test. *p < 0.05, **p < 0.01, ***p < 0.001. (TIFF) [file ppat.1011171.s004.tiff]

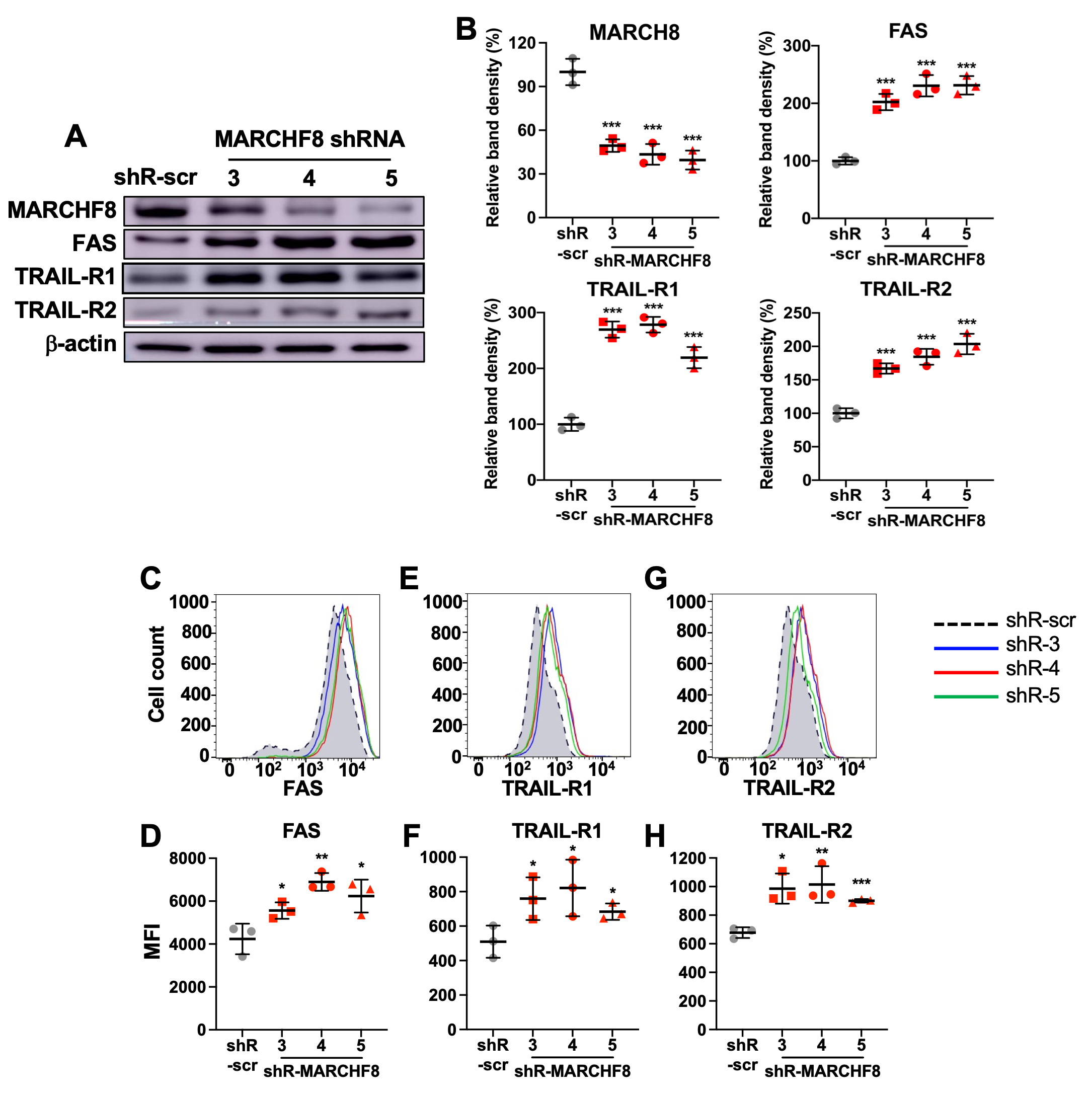

Supplement: S5 Fig — HPV+ HNC (SCC2) cells were transduced with three lentiviral shRNAs against MARCHF8 (shR-MARCHF8 clones 3–5) along with scrambled shRNA (shR-scr). Protein expression of MARCHF8, FAS, TRAIL-R1, and TRAIL-R2 was determined by western blotting (A). Relative band density was quantified using NIH ImageJ (B). β-actin was used as an internal control. The data shown are means ± SD of three independent experiments. Cell surface expression of FAS (C and D), TRAIL-R1 (E and F), and TRAIL-R2 (G and H) proteins were analyzed by flow cytometry. Mean fluorescence intensities (MFI) of three independent experiments are shown (D, F, and H). P values were determined by Student’s t-test. *p < 0.05, **p < 0.01, ***p < 0.001. (TIFF) [file ppat.1011171.s005.tiff]

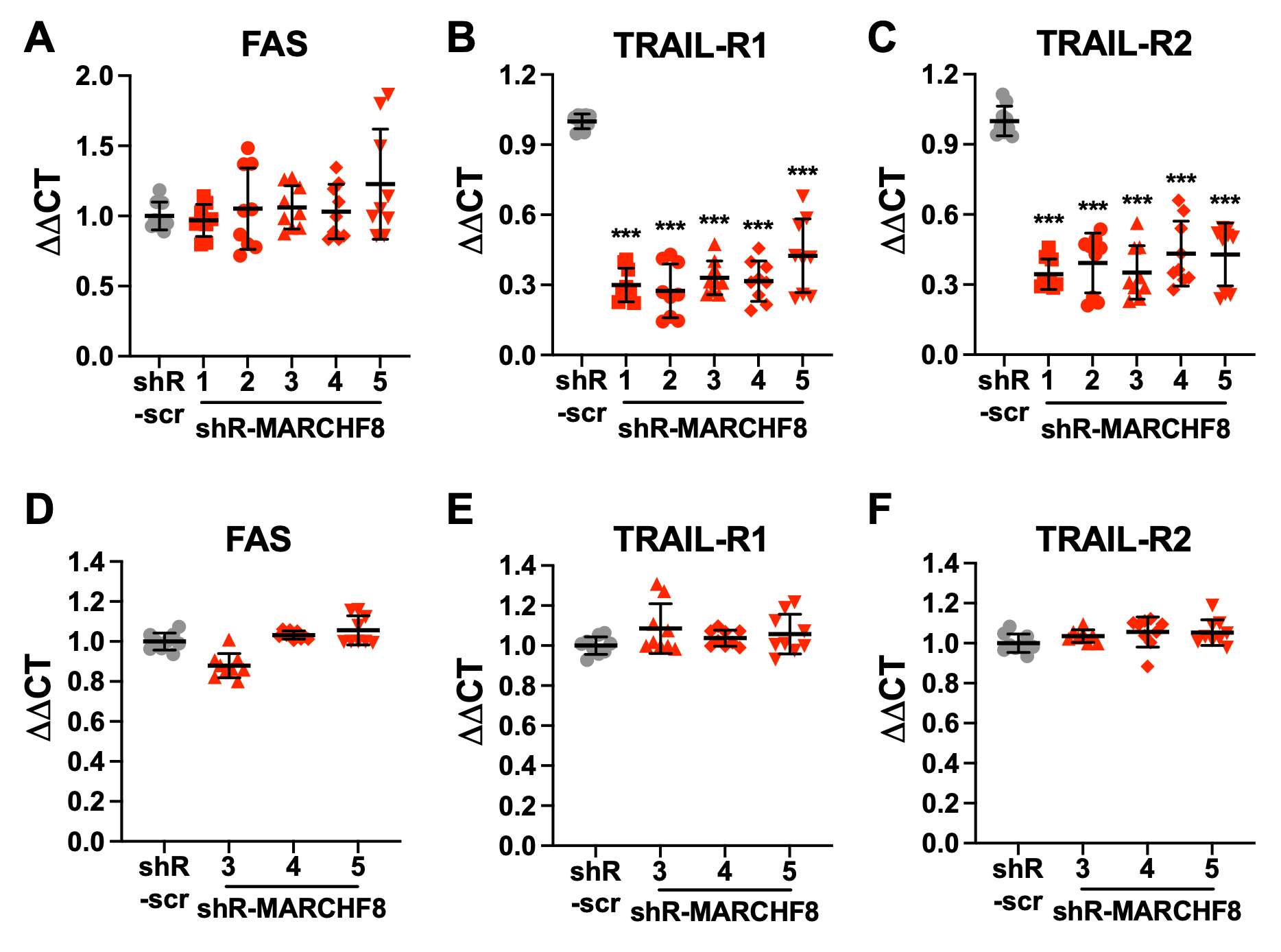

Supplement: S6 Fig — Two HPV+ HNC cell lines, SCC152 (A-C) and SCC2 (D-F) were transduced with five and three lentiviral shRNAs against MARCHF8 (shR-MARCHF8), respectively, or scrambled shRNA (shR-scr). The mRNA levels of FAS (A and D), TRAIL-R1 (B and E), and TRAIL-R2 (C and F) were quantified by RT-qPCR. The data shown are normalized by the GAPDH mRNA level as an internal control. All experiments were repeated at least three times, and the data shown are means ± SD. P values were determined by Student’s t-test. ***p < 0.001. (TIFF) [file ppat.1011171.s006.tiff]

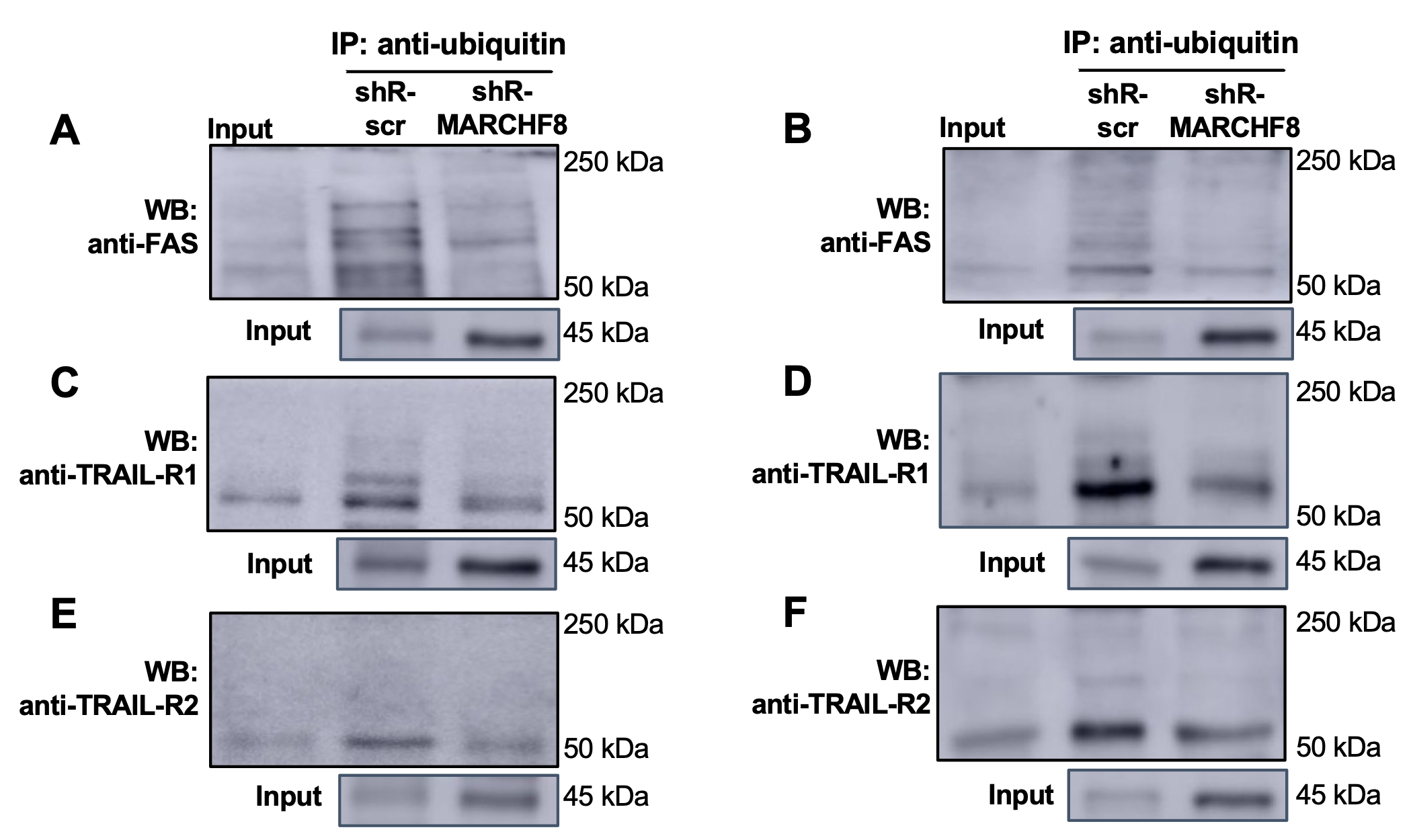

Supplement: S7 Fig — Ubiquitinated proteins were pulled down from the cell lysate of HPV+ HNC (SCC152) cells with scrambled shRNA (shR-scr) or shRNA against MARCHF8 (shR-MARCHF8 clone 3) treated with a proteasome inhibitor MG132 using an anti-ubiquitin antibody (A—F). FAS (A and B), TRAIL-R1 (C and D), and TRAIL-R2 (E and F) proteins were detected in the immunoprecipitated proteins by western blotting. (TIFF) [file ppat.1011171.s007.tiff]
