# Supplementary material for: HPV upregulates MARCHF8 ubiquitin ligase and inhibits apoptosis by degrading the death receptors in head and neck cancer
Source: PLoS Pathog. 2023 Mar 3;19(3):e1011171. doi: 10.1371/journal.ppat.1011171 (PMC10016708; doi:10.1371/journal.ppat.1011171)
Supplement: S1 Table — (PDF) [file ppat.1011171.s008.pdf]

Table S1. MARCHF8 promoter binding proteins

Identified Proteins	Accession Number	Alternate ID	M. Wt	Control 1	Control 2	MARCHF8p 1	MARCHF8p 2
Poly [ADP-ribose] polymerase 1	P09874	PARP1	113 kDa	0	0	1.48E+07	5.89E+07
SAFB-like transcription modulator	Q9NWH9	SLTM	117 kDa	0	0	9938400	8004200
Scaffold attachment factor B1	Q15424	SAFB	103 kDa	0	0	9996200	7113100
Scaffold attachment factor B2	Q14151	SAFB2	107 kDa	0	0	2704600	810820
Heterogeneous nuclear ribonucleoprotein L (Fragment)	A0A3B3ITJ4	HNRNPL	59 kDa	0	0	4042600	2453200
Proliferation marker protein Ki-67	P46013	MKI67	359 kDa	0	0	8838200	47088
Targeting protein for Xklp2	Q9ULW0	TPX2	86 kDa	0	0	6844700	50461
Nucleolar transcription factor 1	E9PKP7	UBTF	87 kDa	0	0	7386700	2329200
Activated RNA polymerase II transcriptional coactivator p15	P53999	SUB1	14 kDa	0	0	3202600	6268700
SNW domain-containing protein 1	G3V3A4	SNW1	65 kDa	0	0	7161900	1230400
Splicing factor 3B subunit 2	Q13435	SF3B2	100 kDa	0	0	7064900	1295700
ATP-dependent DNA helicase 2 subunit 1	B1AHC9	XRCC6	64 kDa	0	0	1.09E+07	2774800
Apoptotic chromatin condensation inducer in the nucleus	E7EQT4	ACIN1	147 kDa	0	0	588660	337190
Thyroid hormone receptor-associated protein 3	A0A3B3ITZ9	THRAP3	104 kDa	0	0	1997000	769640
Transcriptional repressor CTCF	A0A2R8Y595	CTCF	73 kDa	0	0	3623900	385000
Interleukin enhancer-binding factor 2	A0A0A0MRL0	ILF2	16 kDa	0	0	3482900	2894200
pre-rRNA 2'-O-ribose RNA methyltransferase FTSJ3	Q8IY81	FTSJ3	97 kDa	0	0	1859700	18163
RNA helicase	A0A2R8Y4A4	DDX3X	73 kDa	0	0	828180	1123300
RNA helicase	A0A0D9SF53	DDX3X	81 kDa	0	0	828180	1123300
Enhancer of rudimentary homolog	G3V279	ERH	8 kDa	0	0	1996900	4089900
Protein ELYS	Q8WYP5	AHCTF1	253 kDa	0	0	4319800	348800
Paraspeckle component 1	Q8WXF1	PSPC1	59 kDa	0	0	1.19E+07	68122
Polyadenylate-binding protein	A0A087WTT1	PABPC1	65 kDa	0	0	1294000	385710
Serine/arginine-rich splicing factor 5	Q13243	SRSF5	31 kDa	0	0	3408800	2634000
Coiled-coil domain-containing protein 137	Q6PK04	CCDC137	33 kDa	0	0	5973300	264460
Chromatin target of PRMT1 protein	Q9Y3Y2	CHTOP	26 kDa	0	0	3587700	3526100
Zinc finger RNA-binding protein	Q96KR1	ZFR	117 kDa	0	0	1408700	671690
Cysteine and glycine-rich protein 2	Q16527	CSRP2	21 kDa	0	0	2661000	366170
Plectin	Q15149	PLEC	532 kDa	0	0	95971	5216500
Nucleolar RNA helicase 2	Q9NR30	DDX21	87 kDa	0	0	3364500	530030
Poly(rC)-binding protein 1	Q15365	PCBP1	37 kDa	0	0	776010	865050
U5 small nuclear ribonucleoprotein 40 kDa protein	Q96DI7	SNRNP40	39 kDa	0	0	4229400	1028300
CCHC-type zinc finger nucleic acid binding protein	P62633	CNBP	19 kDa	0	0	699640	1069000
Cluster of Polypyrimidine tract-binding protein 1	A0A0U1RRM4	PTBP1	62 kDa	0	0	4603100	652930
RNA-binding protein 15	A0A087WWP4	RBM15	102 kDa	0	0	1942500	498080
Surfeit locus protein 6	O75683	SURF6	41 kDa	0	0	2438200	195930
DNA topoisomerase 2-beta	Q02880	TOP2B	183 kDa	0	0	2149700	833450
Peptidyl-prolyl cis-trans isomerase A	P62937	PPIA	18 kDa	0	0	2300900	1379400
Myc-associated zinc finger protein (Fragment)	I3L2Z5	MAZ	22 kDa	0	0	985790	665450
Zinc finger protein 787	Q6DD87	ZNF787	40 kDa	0	0	1194900	111650
Splicing factor U2AF 35 kDa subunit	Q01081	U2AF1	28 kDa	0	0	5173900	316100
Transcription factor A, mitochondrial (Fragment)	H7BYN3	TFAM	26 kDa	0	0	3675600	931030

U5 small nuclear ribonucleoprotein 200 kDa helicase (Fragment)	A0A494C1A5	SNRNP200	158 kDa	0	0	754300	99806
Heterogeneous nuclear ribonucleoprotein F	P52597	HNRNPF	46 kDa	0	0	835330	143280
Splicing factor U2AF 65 kDa subunit	P26368	U2AF2	54 kDa	0	0	513500	228650
Transcription intermediary factor 1-beta	Q13263	TRIM28	89 kDa	0	0	284780	1237600
Serine/arginine-rich splicing factor 10	O75494	SRSF10	31 kDa	0	0	1968100	494750
PHD finger-like domain-containing protein 5A	Q7RTV0	PHF5A	12 kDa	0	0	470190	405650
Small nuclear ribonucleoprotein E	A6NHK2	SNRPE	6 kDa	0	0	3510700	1085600
Host cell factor 1	P51610	HCFC1	209 kDa	0	0	338640	90807
LINE-1 retrotransposable element ORF1 protein	Q9UN81	L1RE1	40 kDa	0	0	779650	741710
DNA-directed RNA polymerase I subunit RPA34	O15446	POLR1G	55 kDa	0	0	374690	927560
Protein max	G3V5L1	MAX	14 kDa	0	0	733840	2085000
Single-stranded DNA-binding protein, mitochondrial (Fragment)	A0A0G2JLD8	SSBP1	16 kDa	0	0	735890	1503100
Pleiotropic regulator 1	O43660	PLRG1	57 kDa	0	0	697400	232720
ELAV-like protein 1	Q15717	ELAVL1	36 kDa	0	0	545480	112240
Protein RCC2	Q9P258	RCC2	56 kDa	0	0	140180	417960
BUB3-interacting and GLEBS motif-containing protein ZNF207 (Fragment)	J3KS31	ZNF207	35 kDa	0	0	987580	167960
Protein SPT2 homolog	Q68D10	SPTY2D1	76 kDa	0	0	2006200	87993
Plasminogen activator inhibitor 1 RNA-binding protein	Q8NC51	SERBP1	45 kDa	0	0	382320	1415100
Peroxiredoxin-1	A0A0A0MRQ5	PRDX1	11 kDa	0	0	41820	272380
G-rich sequence factor 1	F5H5I6	GRSF1	48 kDa	0	0	78699	117130
PRKC apoptosis WT1 regulator protein	Q96IZ0	PAWR	37 kDa	0	0	3041600	27995
rRNA 2'-O-methyltransferase fibrillarin (Fragment)	M0QXL5	FBL	27 kDa	0	0	494320	754790
GTP-binding nuclear protein Ran	B5MDF5	RAN	26 kDa	0	0	214070	106010
Inner nuclear membrane protein Man1	Q9Y2U8	LEMD3	100 kDa	0	0	212000	108770
Heterogeneous nuclear ribonucleoprotein U-like protein 1	A0A0A0MRA5	HNRNPUL1	86 kDa	0	0	235840	683110
Growth arrest and DNA damage-inducible proteins-interacting protein 1	Q8TAE8	GADD45GIP 1	25 kDa	0	0	145010	231330
Polyglutamine-binding protein 1	O60828	PQBP1	30 kDa	0	0	255690	320530
Exosome RNA helicase MTR4	P42285	MTREX	118 kDa	0	0	67741	244530
Heterogeneous nuclear ribonucleoprotein H (Fragment)	D6R9T0	HNRNPH1	18 kDa	0	0	238970	134110
Upstream stimulatory factor 2 (Fragment)	M0QXT0	USF2	28 kDa	0	0	76741	929030
Transcription elongation regulator 1	A0A7P0T8N8	TCERG1	126 kDa	0	0	52551	119750
Death-inducer obliterator 1	Q9BTC0	DIDO1	244 kDa	0	0	385010	212180
14-3-3 protein zeta/delta (Fragment)	E5RGE1	YWHAZ	6 kDa	0	0	880380	534040
Septin-9	Q9UHD8	SEPTIN9	65 kDa	0	0	43761	157720
Histone-lysine N-methyltransferase EHMT1 (Fragment)	A0A0C4DGF8	EHMT1	88 kDa	0	0	356720	65173
Zinc finger protein 638	Q14966	ZNF638	221 kDa	0	0	149910	253900
Eukaryotic translation initiation factor 4 gamma 1	A0A7I2Y1C3	EIF4G1	173 kDa	0	0	569020	443110
Protein SCAF11	F8VXG7	SCAF11	143 kDa	0	0	128960	130110
Eukaryotic translation initiation factor 2 subunit 3 (Fragment)	H7BZU1	EIF2S3	20 kDa	0	0	180510	75688
La-related protein 1	Q6PKG0	LARP1	124 kDa	0	0	148560	81575
