# Supplementary material for: HPV upregulates MARCHF8 ubiquitin ligase and inhibits apoptosis by degrading the death receptors in head and neck cancer
Source: PLoS Pathog. 2023 Mar 3;19(3):e1011171. doi: 10.1371/journal.ppat.1011171 (PMC10016708; doi:10.1371/journal.ppat.1011171)
Supplement: S2 Table — (PDF) [file ppat.1011171.s009.pdf]

Table S2. List of the antibodies

Antibody	Specificity	Source	Catalog	RRID	Experiment
HPV16 E7 (clone ED17)		Santa Cruz	SC-6981	AB_627745	Western blot
MARCHF8	Human/Mouse	Thermo Fisher	PA5-88893	AB_2805201	Western blot
c-MYC	Human/Mouse	Thermo Fisher	PA5-120193	AB_2913765	Western blot
MAX	Human/Mouse	Proteintech	10426-1-AP	AB_2141660	Western blot
MARCHF8	Human/Mouse	Proteintech	14119-1-AP	AB_2140168	IP
FAS/CD95	Human/Mouse	Proteintech	13098-1-AP	AB_2278042	IP-WB
DR4/TRAIL-R1	Human/Mouse	Proteintech	24063-1-AP	AB_2879421	IP-WB
DR5/TRAIL-R2	Human/Mouse	Proteintech	15497-1-AP	AB_2240702	IP-WB
ubiquitin	Human/Mouse	Proteintech	10201-2-AP	AB_671515	IP-WB
p53	Human/Mouse	Proteintech	10442-1-AP	AB_2206609	Western blot
pRb	Human/Mouse	Santa Cruz	SC-102	AB_628209	Western blot
PE-CD95 (FAS)	Human	BioLegend	305608	AB_314546	Flow cytometry
PE-CD261 (DR4, TRAIL-R1)	Human	BioLegend	307206	AB_2287472	Flow cytometry
PE-CD262 (DR5, TRAIL-R2)	Human	BioLegend	307406	AB_2204926	Flow cytometry
PE-CD95 (FAS)	Mouse	BioLegend	152608	AB_2632902	Flow cytometry
PE-CD262 (DR5, TRAIL-R2)	Mouse	BioLegend	119905	AB_345401	Flow cytometry
CD95 (APO-1/FAS) (15A7)	Mouse	Thermo Fisher	14-0951-85	AB_467393	Apoptosis
CD95 (APO-1/FAS) (EOS9.1)	Human	Thermo Fisher	16-0958-85	AB_469037	Apoptosis
