# Supplementary material for: HPV upregulates MARCHF8 ubiquitin ligase and inhibits apoptosis by degrading the death receptors in head and neck cancer
Source: PLoS Pathog. 2023 Mar 3;19(3):e1011171. doi: 10.1371/journal.ppat.1011171 (PMC10016708; doi:10.1371/journal.ppat.1011171)
Supplement: S3 Table — (PDF) [file ppat.1011171.s010.pdf]

Table S3. List of the shRNAs

Name	Sigma-Aldrich TRC Clone ID	Sequence
Human MARCHF8 shRNA1	TRCN0000073233	5`-CTTGAGCTGAATGAGAGAATA-3`
Human MARCHF8 shRNA2	TRCN0000073234	5`-CCACTAACAGAGCCCAACTTT-3`
Human MARCHF8 shRNA3	TRCN0000073235	5`-CAGTGTAAAGTGTATGTGCAA-3`
Human MARCHF8 shRNA4	TRCN0000073236	5`-CTGGTCCTTGTATGTGCTCAT-3`
Human MARCHF8 shRNA5	TRCN0000073237	5`-CCTCCTTCTCTCGCACTTCTA-3`
