# Supplementary material for: HPV upregulates MARCHF8 ubiquitin ligase and inhibits apoptosis by degrading the death receptors in head and neck cancer
Source: PLoS Pathog. 2023 Mar 3;19(3):e1011171. doi: 10.1371/journal.ppat.1011171 (PMC10016708; doi:10.1371/journal.ppat.1011171)
Supplement: S4 Table — (PDF) [file ppat.1011171.s011.pdf]

Table S4. List of the sgRNAs

Name	Sequence
Mouse MARCHF8 sgRNA1 Fwd	5`-CACCGAGGTGAGTATATGGGCCGTGAGG-3`
Mouse MARCHF8 sgRNA1 Rev	5`-AAACCCTCACGGCCCATATACTCACCT-3`
Mouse MARCHF8 sgRNA2 Fwd	5`-CACCGTATTAACGTCTGACCATGTGAGG-3`
Mouse MARCHF8 sgRNA2 Rev	5`-AAACCCTCACATGGTCAGACGTTAATA-3`
Mouse MARCHF8 sgRNA3 Fwd	5`-CACCGACTACCAGCTTCGTCCAGAAAGG-3`
Mouse MARCHF8 sgRNA3 Rev	5`-AAACCCTTTCTGGACGAAGCTGGTAGT-3`

Fwd, forward oligo; Rev, reverse oligo
