# Supplementary material for: HPV upregulates MARCHF8 ubiquitin ligase and inhibits apoptosis by degrading the death receptors in head and neck cancer
Source: PLoS Pathog. 2023 Mar 3;19(3):e1011171. doi: 10.1371/journal.ppat.1011171 (PMC10016708; doi:10.1371/journal.ppat.1011171)
Supplement: S5 Table — (PDF) [file ppat.1011171.s012.pdf]

Table S5. List of the oligonucleotides

Name	Sequence	Experiment
Human MARCHF8 Fwd	5`-AGTGACATTCCACGTCATTGC-3`	RT-qPCR
Human MARCHF8 Rev	5`-GATCTCCTCAGCAGTACGGTC-3`	RT-qPCR
Human GAPDH Fwd	5`-GGAGCGAGATCCCTCCAAAAT-3`	RT-qPCR
Human GAPDH Rev	5`-GGCTGTTGTCATACTTCTCATGG-3`	RT-qPCR
Human FAS Fwd	5`-TTGCTGCCATCTGGTAGTGTG-3`	RT-qPCR
Human FAS Rev	5`-TGGTGCAGTGAATGCTCGAAG-3`	RT-qPCR
Human TRAIL-R1 Fwd	5`-ACCTTCAAGTTTGTCGTCGTC-3`	RT-qPCR
Human TRAIL-R1 Rev	5`-CCAAAGGGCTATGTTCCCATT-3`	RT-qPCR
Human TRAIL-R2 Fwd	5`-GCCCCACAACAAAAGAGGTC-3`	RT-qPCR
Human TRAIL-R2 Rev	5`-AGGTCATTCCAGTGAGTGCTA-3`	RT-qPCR
MARCHF8 promoter +160 Rev	5`-AAGCTTTCCTAGAGGCCGCAGGAGTTACCTCAGGTG-3`	Cloning
MARCHF8 promoter -1340 Fwd	5`-GGTACCGGCAACTGGCCTTGATAACCTATTTTCCAC-3`	Cloning
MARCHF8 promoter -840 Fwd	5`-GGTACCCATGCCTGTATTCCCAGCACTTTGGCAGGC-3`	Cloning
MARCHF8 promoter -90 Fwd	5`-GGTACCTGCAGCCCGCCCCCAGCGCGCTGCAGTCGCCG-3`	Cloning
MARCHF8 promoter -60 Fwd	5`-GGTACCCGGGGCAACGCCTCCACCCAACTCGGCCGGACA-3`	Cloning
MARCHF8 promoter -30 Fwd	5`-GGTACCACACGTGACGCCCGCGTCACGTGACCTGCCACT-3`	Cloning
MARCHF8 promoter -10 Fwd	5`-GGTACCGTGACCTGCCACTCGGTGTCCTCCCGCGGAGGG-3`	Cloning
MARCHF8 promoter E-box1 mutant Fwd	5`-TCGGCCGGACACGTGACGCCCGCGTCATGACCTGCCACTCGGTGT-3`	Cloning
MARCHF8 promoter E-box1 mutant Rev	5`-ACACCGAGTGGCAGGTCATGACGCGGGCGTCACGTGTCCGGCCGA-3`	Cloning
MARCHF8 promoter E-box2 mutant Fwd	5`-ACTCGGCCGGACATGACGCCCGCGTCACGTGACCTGCCACTCGGT-3`	Cloning
MARCHF8 promoter E-box2 mutant Rev	5`-ACCGAGTGGCAGGTCACGTGACGCGGGCGTCATGTCCGGCC GAGT-3`	Cloning
MARCHF8 promoter E-box1/2 mutant Fwd	5`-ACTCGGCCGGACATGACGCCCGCGTCATGACCTGCCACTCGGTGT-3`	Cloning
MARCHF8 promoter E-box1/2 mutant Rev	5`-ACACCGAGTGGCAGGTCATGACGCGGGCGTCATGTCCGGCC GAGT-3`	Cloning

Fwd, forward primer; Rev, reverse primer
