# Supplementary material for: HPV upregulates MARCHF8 ubiquitin ligase and inhibits apoptosis by degrading the death receptors in head and neck cancer
Source: PLoS Pathog. 2023 Mar 3;19(3):e1011171. doi: 10.1371/journal.ppat.1011171 (PMC10016708; doi:10.1371/journal.ppat.1011171)
Supplement: Attachment — Submitted filename: Response-Khalil.pdf [file ppat.1011171.s013.pdf]

Response to Editor and Reviewers

We are grateful to the reviewers for recognizing our study as “well done, clearly written and will contribute importantly to understanding virus/host interactions,” “significant work,” “of high rigor,” “The data strongly supports their conclusion,” and “well-conceived and well-executed work,” and for providing useful comments that have helped us improve our manuscript. In response to the reviewers’ comments point by point, we have revised our manuscript as described below:

Editor

Comment 1: First, contrary to the title, the data show that both E6 and E7 can increase levels of MarchF8. This needs to be addressed and mutants of E6 should be use to better address the mechanism.

Response: We agree and have changed the title and the text (Lines 40, 78, 336-337) to include both E6 and E7 as MARCHF8 inducers. We also determined the expression levels of MARCHF8 mRNA and protein in the N/Tert-1 cell lines that express E6 8S9A10T (deficient in p53 binding), E6 I128T (deficiency in E6AP binding), or E7 ΔDLYC (deletion of the pRb binding domain). The results showed that all these mutants upregulate MARCHF8 mRNA and protein expression to similar levels of the wild type E6 and E7, suggesting that p53 and pRb degradation is unnecessary for MARCHF8 upregulation by E6 and E7 (**Fig. S2A-2D**). To further analyze promoter activity, we cotransfected HPV- HNC cells (SCC1) with the MARCHF8 promoter reporter plasmid and either E6 Y54D (deficient in p53 binding), E6 I128T (deficient in E6AP binding) or E7 ΔDLYC (deletion of the pRb binding domain) and measured luciferase activity. Consistent with the RT-qPCR data (**Fig. S2A**), both E6 Y54D and E6 I128T expression significantly induced MARCHF8 promoter activity comparable to wildtype E6 expression (**Fig. S3B**). Thus, to fully understand the mechanism of how E6 induces MARCHF8 promoter, an extensive investigation would be necessary using various E6 mutants. In addition to new figures (**Figs. S2A and S3**), these new results are added in the Results and Discussion sections (Lines 109-116, 134-145, 367-369).

Comment 2: Second, one reviewer points out that the difference in expression of MARCHF8 comparing HPV + versus HPV- in head and neck tumors is relatively small and could be explained by a number of other factors. These should be included in interpreting the results.

Response: We appreciate this important point of MARCHF8 expression comparing HPV+ and HPV- HNCs. We noticed that MARCHF8 mRNA levels in HPV- HNC patients are highly variable compared to MARCHF8 mRNA levels in HPV+ HNC patients in **Fig. 1A**. This may indicate that the expression of MARCHF8, one of the various factors to inhibit cancer cell apoptosis, could be regulated by more diverse cellular factors in HPV- HNC compared to HPV+ HNC in which HPV is a dominant factor. The difference in MARCHF8 expression levels between HPV+ and HPV- HNCs is now discussed in the Discussion section (Lines 377-385).

Comment 3: Third, Multiple replicates of the MARCHF8/Fas/Ub (and other death receptor) complex pulldowns in Fig 5 should be quantified.

Response: We agree with the reviewer and quantified three replicates of the pulldown assays for the MARCHF8 protein complex with FAS, TRAIL-R1, and TRAIL-R2. The quantification data graphs (**Fig. 5F-5H**) and their original blots (**Fig. S7A-S7F**) are now added.

Reviewer #1

Comment: Its puzzling that E7 causes increased steady state levels of MARCHF8 transcript but has no effect on MARCHF8 transcription in the promoter assays. Is E7 known to modulate RNA stability of other cellular transcripts? Could their be additional E7-responsive elements further than 1kb upstream of the MARCHF8 ORF? Might E7 have an effect if the promoter assays are done in primary keratinocytes?

Response: We thought these were interesting ideas and further investigated the reviewer's hypothesis. To test if E7-responsive elements reside further than 1 kb upstream of the MARCHF8 ORF, we cloned extended MARCHF8 promoter regions up to 1.5 kb upstream of the MARCHF8 ORF and performed the promoter-reporter assay. The results showed that E7 did not induce the immediate upstream promoter up to 1.5 kb. These results and the sequence of the 1.5 kb MARCHF8 promoter we tested are now available in **Fig. S3A and S3B**, respectively. As far as we know, no published data have shown that E7 modulates the RNA stability of any cellular transcripts. Nevertheless, we do not exclude the possibility of MARCHF8 mRNA stabilization by E7 and will consider investigating it in future. All these points are now discussed in the Discussion section (Lines 377-380).

Reviewer #2

Comment 1: The authors have examined previously acquired RNA expression data derived from microarray hybridizations for the expression of MARCHF8 ubiquitin ligase expression in head and neck cancers. Expression of MARCH8 was compared between multiple cancer samples that were either HPV+ or HPV- or to multiple samples of normal epithelium (site not otherwise specified). They observed modestly elevated expression of MARCH8 RNA in the HPV+ compared to the normal epithelium (Fig 1A). Although the large number of samples allows for statistical significance, it is likely that most of the normal epithelial sample is comprised of differentiated cells that may have lower MARCH8 expression compared to proliferative basal cells

Response: We thought this was an insightful point. To test if differentiated cells have lower MARCHF8 expression than proliferative basal cells, we analyzed the MARCHF8 expression from the same dataset comparing poorly/undifferentiated and well/moderately differentiated HNC samples, based on the histological grade [PMID: 17510386, **Table S1**]. Our analyses showed no significant difference in MARCHF8 expression in HPV+ HNC alone and combined HPV+ and HPV- HNC samples (Figure on the right). These results suggest that differentiation status might not affect MARCHF8 mRNA levels in HNC patients. Additionally, our MARCHF8 expression data from the cultured cells also support that HPV is the main determinant of increased MARCHF8 expression in HNC.

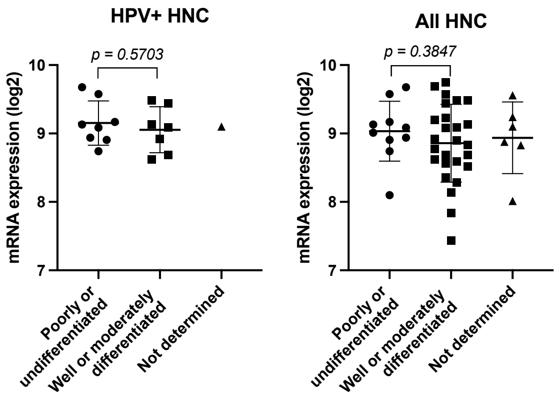

Comment 2: There was no statistical difference between either the normal to HPV- sample sets or between the HPV+ and HPV- cancer sets... Another way to demonstrate specificity would be the use of mutants of E6 and E7 which is not done.

Response: Please see our responses to *Editors' Comments 1 and 2* above.

Comment 3: The comparison between HPV+ and HPV- cancers for MARCH8 expression is less compelling because we do not know if these two types of cancers even originate from the same epithelial cell type, meaning that other reasons, not contemplated by the authors, could account for the difference in MARCH8 expression between HPV+ and HPV- cancer cells.

Response: We agree with the reviewer that MARCHF8 expression can be regulated by other mechanisms in HPV- HNC, while the HPV oncoproteins are mainly responsible for MARCHF8 upregulation in HPV+ HNC. It may be the reason why the MARCHF8 expression levels in HPV- HNC are highly variable compared to HPV+ HNC and normal samples. Please see our responses to *Comment 1* and *Editor's Comment 2* above for details.

Comment 4: Fig.2 demonstrates an effect between E-boxes and E6 induction of a synthetic promoter construct. Yet the effect is not compelling in that upon mutation of both E-boxes in the reporter plasmid, E6 activates the promoter 20X instead of 40X. That may be significant, but is it important? Given that they show E7 activates MARCH8 at the promoter level as well as E6 in TERT cells, I do not think Fig. 2 contributes to the arguments they are making.

Response: Although E7 expressing N/Tert-1 cells also show a high MARCHF8 mRNA level, it may be caused by other mechanisms, such as increased mRNA stability, not promoter activation. We performed new experiments to further analyze potential mechanisms according to Editor's recommendation. Details are available in our responses to *Editors' Comments 1* above

Comment 5: In Fig. 3, the SCC2 cell line seem non-supportive of their hypothesis for a role of E6 and E7 in that while MARCH8 levels are high (from Fig. 1) TRAILR1 levels are not reduced. The more informative experiment is the transduction of tert immortalized cells with viral oncoproteins and FACS analysis of death receptor expression. In this case, E6, E7, and E6/E7 all reduce surface expression. E6 does not reduce levels seen in cells also transduced by E7. This is inconsistent with their hypothesis of specificity of action of E6 vs E7 and supports a non-specific mechanism at work in oncogene expressing tert cells. Again, a careful mutant analysis might support a specific mechanism that is not obvious as yet.

Response: Please see our response to *Editor's Comments 1*.

Comment 6: In Fig 4 the authors set up a crucial experiment, using shRNA to knock down MARCHF8 expression. It is a bit odd that all 5 shRNA knock down MARCHF8 to a similar degree, which has almost never been my experience with a set of 5 shRNA lentiviruses. This raises the possibility that the expression difference between scramble and the 5 shrna to MARCH8 is a difference that could be attributed to the scramble control. Expression of an shRNA escape MARCHF8 mRNA might resolve this possibility. The data does show that compared to scramble all 5 shRNA reduce MARCHF8 and increase death receptor expression as noted by the authors.

Response: We agree that it is unusual to see similar knockdown levels from 5 different shRNAs. However, we used the same shRNAs in another HPV+ HNC cell line (SCC2), in which shRNA clones 4 and 5 clearly showed better knockdown than clone 3, which correlate to the increase of FAS and TRAIL-R2 protein levels (**Fig. S5A and S5B**). Further, we showed FAS, and TRAIL-R1/2 upregulation by Marchf8 knockout using sgRNAs in mouse HPV+ HNC cells (**Fig. 8A and 8B**). As all experiments of MARCHF8 knockdowns with shRNAs and knockouts with sgRNAs in multiple human and mouse HPV+ HNC cells consistently show the same results, it is unlikely that "the expression difference between scramble and the five shRNAs to MARCHF8 is a difference that could be attributed to the scramble control."

Comment 7: In Fig. 5c, which band is Ub-Fas? Overall, Fig. 5 is supportive of MARCHF8 having a role in the ubiquitination of Fas, Trail's and thereby negative regulation. This corroborates earlier results from others.

Response: Ubiquitinated proteins are normally shown as multiple bands due to different sizes from the different numbers of ubiquitins conjugated to the target protein. Thus, all of the bands in **Fig. 5C** must be the unmodified and ubiquitinated forms of FAS protein.

Comment 8: Fig 6 supports the conclusion that when Marchf8 expression is manipulated by shRNA, apoptoBc traits are altered as expected.

Response: Thank you for your agreement.

Comment 9: Fig 7 utilizes a mouse expression system comparing "normal" mouse oral epithelium, the same cells transformed with HPV and the same cells transformed with HPV. Here the data is less

convincing. Starting cells are compared to HPV-transformed (not specified if retrovirus, plasmid, HPV genomes or what) and HPV-non-transformed cells (oncogenes used in the transformation unspecified). So we have no idea what mechanisms might be involved. But what is clear is that both types of transformation events induced the expression of marchf8, with fas and trail-r1 alteration in the expected pattern. However, and unexpectedly, Trail-2 was not reduced in HPV- transformants but decreased in HPV transformants.

Response: As mentioned in our response to Reviewer 1's Comment, we agree that the mechanism of MARCHF8 upregulation in some HPV- HNC cells is unclear. While no animal model can perfectly recapitulate HPV infection oncogenesis in human patients, the mEERL syngeneic model [PMID: 19917928] is well accepted in the field as a useful HPV+ HNC mouse model [PMIDs: 33986123, 31627350, 24700450, 34230113, 35725568, 29313973, 28811971, 31409394, 32391119, 34703882...]. We already described cell line information as "we adopted a mouse model of HPV+ HNC with *Hras*-transformed mouse oral epithelial cells expressing HPV16 E6 and E7 (MOE/E6E7 or mEERL) that form tumors in immunocompetent syngeneic C57BL/6 mice [30]. ... compared to ... mouse MOE cells transformed with *Hras* and shR-Ptpn14 (HPV- MOE)" (Lines 270-275). As the reviewer pointed out, the decrease of TRAIL-R2 expression in HPV- HNC cells is consistently not as significant as in human and mouse HPV+ HNC cells. Understanding potential mechanisms, such as the role of unidentified E2 ubiquitin-conjugating enzymes, requires further investigation.

Comment 10: In Fig. 8, marchf8 is knocked out using crispr and marchf8, fas, trail-r1 and trail-r2 expression follows the expected pattern. In Fig. 9 knockout of marchf8 reduces tumor growth rate in HPV expressing cancer cells, as one might expect from purported effects upon apoptosis, but also possibly from march8 effects upon other as yet undescribed effects upon other cellular transmembrane proteins.

Response: We fully agree.

Reviewer #3

Minor Comment 1: The manuscript demonstrates that following E6 expression, FAS, TRAIL-R1 and TRAIL-R2 are targets of MARCHF8 for ubiquitinylation and degradation. Are these proteins considered to be natural or canonical targets of MARCHF8, or is this enhanced turnover a specific consequence of the virus?

Response: FAS, TRAIL-R1, and TRAIL-R2 are likely natural targets, as another study showed that MARCHF8 expression downregulates TRAIL-R1 independently of virus infection [PMID: 23300075]. However, it is mostly unknown about the detailed mechanism including corresponding E2 ligases and cofactors in the ubiquitin ligase complex(es) that target FAS, TRAIL-R1, and TRAIL-R2.

Minor Comment 2: The authors state (line 51) that HPV is associated with about 25% of head and neck cancers, with no reference given. Estimates vary, but most recent estimates put the percentage of HN cancers that are HPV+ to be significantly higher than 25%.

Response: Thank you for noticing the missing references. Estimates indeed vary. HPV-positive cases are much higher in the oropharyngeal region compared to other regions. However, HPV-positive cases among all head and neck cancers are currently estimated as 25%. We added three references accordingly.

Minor Comment 3: The authors note that both E6 and E7 can upregulate expression of MARCHF8, but found that when using the plasmid reporter system, only E6 gave a positive signal. They then note that there may be an alternative mechanism associated with E7. It would have been helpful, perhaps in the discussion, to speculate on what some possible mechanisms might be.

Response: Please see our response to *Editors' Comment 2*.

Minor Comment 4: When assessing apoptosis, the authors used FasL and anti-Fas to induce the process. It would have been interesting to see the impact of modulating MARCHF8 expression on apoptosis induced through other receptors (like TRAIL or TNF), or even through a pathway that routes through p53. This would have helped to further define the scope of this particular MARCHF8-dependent mechanism.

Response: We agree with the reviewer that expanding the scope MARCHF8-dependent mechanism by testing other pathways, such as TRAIL-R1/2 and p53, will provide interesting and useful information. We will consider continuing our investigation into these aspects.

Minor Comment 5: In the mouse oral cancer cells, while there is data provided on surface expression of Fas and TRAIL-R2, no data is given for TRAIL-R1. The total level of TRAIL-R1 is addressed.

Response: We could not examine the surface expression of TRAIL-R1 on mEERL cells, as a fluorophore-conjugated mouse TRAIL-R1 antibody for flow cytometry was unavailable. We added this point in the Results section for clarification (Lines 296-299).

Minor Comment 6: The authors note the death domain carried by the target proteins. What other proteins carry this same domain, and is it anticipated that some or all of them might also be targets for MARCHF8?

Response: In addition to FAS (CD95), TRAIL-R1, and TRAIL-R2, several other TNFRSF receptors, such as TNFR1, TNFRSF16, and TNFRSF21, have death domains. FAS, TRAIL-R1, and TRAIL-R2 have been found as key apoptosis regulators targeted by membrane-associated ubiquitin ligases. However, it is still possible that other TNFRSF death receptors are substrates of MARCHF8.

Minor Comment 7: Figure 10 is a helpful schematic. However, it is not referred to in the text.

Response: Thank you for picking up this omission. We added it to the citations in the Discussion section (Line 338).

Other Changes

• We added Harrison Nabors as a co-author in the revised manuscript. He helped perform RT-qPCR for the additional experiments suggested by reviewers.

• The Author Contributions section has been reorganized according to the journal guideline.

• As PLoS journal does not allow "data not shown" as a reference, we added the data of apoptosis stimulated by anti-FAS in **Fig. 6**.
